# The Effects of Temperature on Political Violence: Global Evidence at the Subnational Level

**DOI:** 10.1371/journal.pone.0123505

**Published:** 2015-05-20

**Authors:** Alexander Bollfrass, Andrew Shaver

**Affiliations:** Woodrow Wilson School of Public and International Affairs, Princeton University, Princeton, NJ, U.S.A.; University of Washington, UNITED STATES

## Abstract

A number of studies have demonstrated an empirical relationship between higher ambient temperatures and substate violence, which have been extrapolated to make predictions about the security implications of climate change. This literature rests on the untested assumption that the mechanism behind the temperature-conflict link is that disruption of agricultural production provokes local violence. Using a subnational-level dataset, this paper demonstrates that the relationship: (1) obtains globally, (2) exists at the substate level — provinces that experience positive temperature deviations see increased conflict; and (3) occurs even in regions without significant agricultural production. Diminished local farm output resulting from elevated temperatures is unlikely to account for the entire increase in substate violence. The findings encourage future research to identify additional mechanisms, including the possibility that a substantial portion of the variation is brought about by the well-documented direct effects of temperature on individuals' propensity for violence or through macroeconomic mechanisms such as food price shocks.

## Introduction

Prior demonstrations of an empirical relationship between higher temperatures and political violence have been sensitive to measurement strategies and have not provided evidence of a causal mechanism. This paper establishes that the relationship obtains globally when measured at the provincial level and is not geographically restricted. It also tests the widely held assumption that the causal relationship is mediated through a local decline in agricultural production. The finding that the relationship exists even in areas of the world without croplands should direct further investigations away from this assumption. These results contribute empirically and theoretically to the growing literature on political and social effects of changes in the earth’s climate.

The empirical relationship between higher temperatures and increased substate violence has been demonstrated in many settings. A recent meta-analysis of 60 prior studies finds substantial effects of temperature increases on the likelihood of interpersonal and intergroup conflict [1]. Similar associations with changes in precipitation patterns have also been identified. This paper holds precipitation fixed as a means of directly assessing the temperature-conflict relationship.

Such reported temperature-conflict correlations have been met with skepticism on the basis that they either are spurious or suffer from omitted variable bias. Furthermore, the mechanisms that would explain the conditions under which variations in temperature predict violent behavior have not been rigorously investigated. In the extant literature, the causal mechanism is undertheorized and rests heavily on the assumption that local agricultural output is the crucial mediating variable.

## Temperature and civil war in sub-Saharan Africa

The most thorough scholarly exchange to date has centered on Burke et al.’s 2009 finding that hotter annual temperatures have led to increased civil war incidence in sub-Saharan African states [2]. This area has been the primary geographic focus, given policy concerns over the region’s vulnerable populations and “heavy dependence on rainfed agriculture” [3]. Critical rejoinders highlight that this conclusion may be sensitive to measurement, dataset selection, and methodological strategies [4, 5]. Specifically, responses to Burke et al. have centered on four primary criticisms, as articulated by Buhaug [4, 6]. First, the sub-Saharan geographic scope excludes potentially relevant cases of violence in Africa’s Sahel region. Second, the dependent variable is measured with an excessively restrictive per year battle death threshold. Buhaug notes as a third concern that the two decades examined by Burke et al. saw simultaneous warming and conflict. Since the turn of the millennium, temperatures have continued their rise, while conflict has become less prevalent on the continent. Finally, he criticizes Burke et al.’s use of country fixed effects. Additional skepticism relates to the undertheorization of the causal mechanism and insufficient attention to covarying “geopolitical and social factors” [7].

In response to these criticisms, Burke et al. revised their model and generated additional results confirming that variation in the incidence of large wars in sub-Saharan Africa in the 1980s and 1990s is in part explained by temperature change [8]. The updated finding does not hold for the 2003 to 2008 period, which the authors argue may be the result of economic development, improvements in domestic governance, or international peacekeeping efforts [8].

## Identifying the path to conflict

Most previous studies have theorized that the effect of climate on conflict operates through local economic conditions. The first step in this chain of causation is that higher temperatures depress agricultural output. Within Africa, this effect is well established: “[t]emperature can affect agricultural yields both through increases in crop evapotranspiration (and hence heightened water stress in the absence of irrigation) and through accelerated crop development, with the combined effect of these two mechanisms often reducing African staple crop yields by 10%–30% per °C of warming” [9].

Diminished agricultural yield is then theorized to drive conflict by affecting local labor markets and socioeconomic cleavages: young men are “more likely to take up arms when income opportunities are worse for them in agriculture […] relative to their expected income as [fighters]” [10]. Agricultural shocks may also “produce greater income inequality, [heightening] resentment and [generating] tensions across social classes” [10]. From this theoretical perspective, subsequent studies have sought to predict the consequences of climate change on violence levels by extrapolating from historical temperature and rainfall trends [3, 11–13].

The near-exclusive focus on sub-Saharan and Sahelian regions limits the inferences that can be drawn about the temperature-conflict relationship. While localized economic effects of diminished farm output may increase conflict likelihood, other areas of research give reason to believe that rising temperatures can increase that likelihood through channels not specific to those regions of the continent.

Temperature-induced variation in agricultural yield can alter migration patterns, with potential effects on substate violence [14]. Research shows a “significant effect of climate-driven changes in crop yields on the rate of [Mexican] emigration to the United States” [15]. Conflict research also links migrants to the spread of civil war. They “may facilitate the transnational spread of arms, combatants, and ideologies conducive to conflict[,] alter the ethnic composition of the state, [and] exacerbate economic competition” [16].

Excessive heat can reduce the supply of crops, which in most circumstances raises the price of food. In turn, higher food prices have been shown to lead to social instability, including incidents of group violence [17]. However, the “causal link between food insecurity and civil conflict is complex” and depends on a set of economic and political dimensions [18]. The association may exist only in low income countries and have changed fundamentally since the end of the Cold War [19]. The globalization of food prices provides a transmission mechanism from ambient temperature variation in one location to fluctuations in violence in others.

Temperature’s effect on economic activity is also not restricted to agriculture and includes industrial production and political stability. Within the world’s poor countries, economic output and growth diminish with increased temperature, with an estimated effect of a 1.3 percentage point reduction in GDP with every 1°C increase [20]. These effects have been shown to increase a country’s vulnerability to coups d’état [20]. Other studies have observed similar contractions in industrial output [21, 22].

Finally, empirical psychological research has established the tendency of individuals to behave more violently at higher temperatures, leaving “little doubt or controversy about the existence of a heat-violence relation in real-world data” [23]. Relying on the records collected by law enforcement agencies, several robust analyses have found that much variation in violent offenses can be explained by temperature change [23, 24]. In post-conflict environments where peace is tenuous, temperature induced aggression may be sufficient to trigger escalatory violence, leading to renewed fighting.

A logical next step in the research agenda involves testing whether previous results linking temperature and conflict obtain globally. A second step is to determine whether empirical evidence exists in favor of causal mechanisms aside from the localized agricultural effects already identified within the sub-Saharan/Sahelian Africa context.

Before carrying out these tests, this paper addresses two methodological issues found within the existing literature on the linkage between temperature and conflict. First, in the absence of spatially disaggregate data, previous research has adopted the country-year as the unit of statistical analysis by necessity. However, measuring micro relationships at this level may result in inconclusive findings even where relationships between variables exists because of insufficient statistical power. Worse, coefficients generated this way may suffer from aggregation bias, a possibility when inferences are drawn from the broader population to which the unit of interest belongs [25].

Second, existing research has tended to overlook a set of potentially significant variables, whose absence from previous statistical tests may have introduced bias. Existing research supports the premise that insurgent violence is more likely to emerge and persist in environments in which terrain encumbers more powerful counterinsurgents. Countries with a higher proportion of mountainous terrain and forest cover, for instance, are more likely to experience civil war onset and conflict of greater duration, respectively [26, 27]. If temperature is correlated with regions of the world dense in terrain types favorable to insurgency, then the results generated in prior research would likely be biased.

## Results

**Fig 1:**
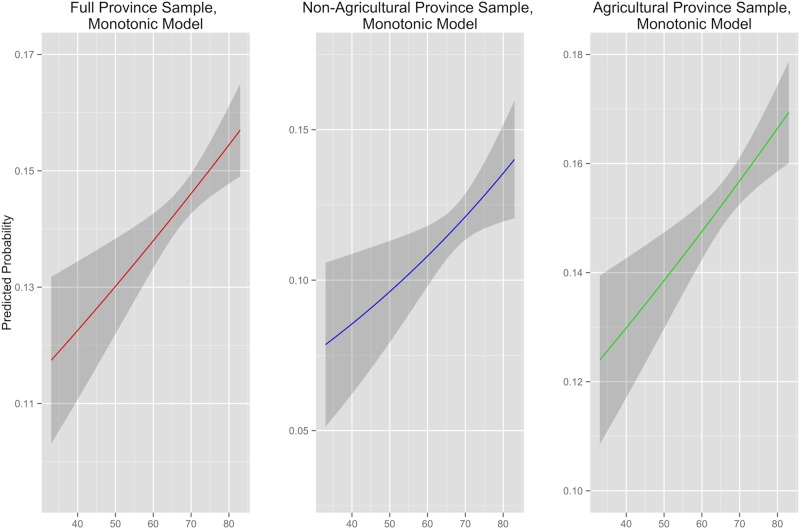
Predicted Probability of Conflict and Yearly Average Temperature, with 95% Confidence Intervals. Agriculture and Non-Agriculture Sample Provinces Compared

**Fig 2:**
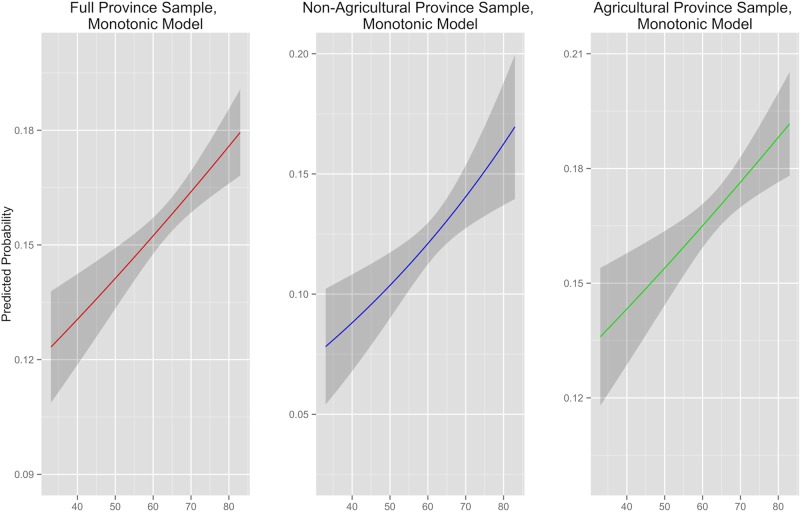
Non-Africa Subset: Predicted Probability of Conflict and Yearly Average Temperature, with 95% Confidence Intervals. Agriculture and Non-Agriculture Sample Provinces Compared

Absolute temperature level is significantly and positively correlated with incidence of conflict around the world (Table 1, column 1 and Fig 1, column 1). This relationship manifests in both non-agricultural and agricultural sample provinces (Fig 1, columns 2 and 3, respectively). Over the entire sample, a difference in mean annual temperature of 20 °F is associated with an approximate 2% change in the likelihood of deadly conflict. Within a given country, the predicted probability of conflict varies significantly over large temperature differences, while incremental temperature change is associated with modest average increases in conflict likelihood. Between the disjoint agricultural and non-agricultural subsets, the predicted increase in conflict likelihood with greater ambient temperature is greater in the former than the latter (Table 1, columns 2 and 3 and Fig 1, columns 2 and 3). Figs 1 and 2 express predicted probabilities generated with models results and should not be confused with graphical representations of the logistic function. Visual similarities are coincidental.

**Table 1:** Incidence of Substate Violence on Temperature (Conditional Logistic Regression).

	*Dependent Variable: Civil War Incidence*		
	(Full Sample)	(Non-Agricultural Province Subset)	(Agricultural Province Subset)
Temperature	0.076 (0.011)***	0.195 (0.040)***	0.077 (0.012)***
Precipitation	0.013 (0.003)***	0.005 (0.013)	0.015 (0.004)***
Distance to Foreign Border	−1.136 (0.410)**	0.683 (1.790)	−1.785 (0.464)***
Distance to Capital	−0.085 (0.134)	−0.706 (0.336)*	0.124 (0.165)
Province Size	−0.004 (0.001)***	0.003 (0.001)	−0.006 (0.001)***
Population (log)	0.517 (0.055)***	0.528 (0.188)**	0.509 (0.063)***
Observations	26,477	6,802	19,489
R^2^	0.018	0.018	0.021
Max. Possible R^2^	0.110	0.055	0.111
Log Likelihood	−1,304.678	−132.761	−941.879
Wald Test (df = 13)	360.190***	62.180***	291.310***
LR Test (df = 13)	470.567***	120.819***	403.680***
Score (Logrank) Test (df = 13)	427.560***	96.512***	353.911***

*p < 0.05;

**p < 0.01;

***p < 0.001

NOTE—All regressions include terrain and ethnic group controls and country-year fixed effects.

## Discussion

The link between higher temperature and violent substate conflict is a global phenomenon not restricted to particular territories within Africa. The statistical relationship between temperature and incidence of conflict is significant and robust to a set of potentially confounding variables absent in prior research. Within each year of the study period, provinces with higher ambient temperatures tended to experience conflict more often than counterpart provinces of same country with lower temperatures. The results predict that areas of the world with higher future average ambient temperatures are more likely to experience civil conflict. Whether this is the result of fundamental differences between warmer and cooler provinces or of temperature changes within individual provinces (or both) is uncertain. In either case, these net temperature effects are likely to vary in intensity with geography and through interactions with other variables, such as precipitation.

The results cast doubt on the assumption undergirding previous research that the relationship is channeled primarily through local effects resulting from changes in agricultural yield. First, violence can be predicted with mean temperatures in provinces that do not experience fluctuations in agricultural output because they had no croplands to begin with. Second, the magnitude of the temperature’s effect on predicted conflict probability is no greater in agricultural areas of the world than in non-agricultural provinces.

The possibility that temperature can be correlated with conflict in non-agricultural settings through spillover violence from affected adjacent agricultural provinces is highly unlikely. First, the unit of analysis is the province-year. Second, provinces identified as hosting camps for either refugees or internally displaced persons are excluded from the analysis. Spillover violence could therefore account for the results only if agriculture-mediated conflict travels over significant distances and is the result of some process other than migration patterns initiated by climate change. Plausible alternative mechanisms through which agriculturally induced violence might travel are absent from the extant literature. Perhaps most importantly, spillover violence would not account for the difference in the functional form describing the temperature-violence relationship between agricultural and non-agricultural areas of the world.

These findings should motivate future research into mechanisms that do not rely on this linkage. The findings are also generally consistent with expectations established by prior psychological findings that temperature affects individuals directly. The vast literature on the physio-psychological effects of heat on the individual’s propensity for violence would be a fruitful resource to initiate such an investigation into non-agricultural mechanisms. Prior research has also identified the transmission of higher food prices as a separate possible mechanism that retains agricultural origins, but whose effects may not manifest near the temperature-damaged crops.

## Materials and Methods

Georeferenced data on conflict, temperature, agriculture, migrant populations, and other pertinent covariates generated in recent years make it possible to analyze the temperature-conflict relationship in greater detail. In particular, land cover data generated at a spatial resolution of 300 meters using a medium resolution imaging spectroradiometer (MERIS) sensor sensitive to vegetation type allows for the disaggregation of subnational units into agricultural and non-agricultural areas [28].

If temperature change influences conflict likelihood, more substate violence should be observed where temperatures are higher. Furthermore, if increased temperature serves to increase conflict likelihood exclusively through localized effects of agricultural production, conflict should not covary with temperatures in areas of the world that do not produce crops.

To test these hypotheses, conditional logistic regression is used first to generate an association between annual incidence of substate conflict and temperatures for every first-order internal administrative boundary (state, province, governorate, etc.) in the world over the past two decades for which temperature and precipitation data is available. First, the following equation is tested:

$$P\left(Y_{t,i}=1|T_{t,i},P_{t,i},S_{t,i}\right)=logit^{-1}\left(\alpha_{i}+\beta T_{t,i}+\delta P_{t,i}+\xi S_{t,i}+\varrho_{t,k}\right)$$ (1)

where *t*, *i*, and *k* denote time periods {1, …, *m*}, subnational units {1, …, *n*}, and counties {1, …, *p*}, respectively.

Incidents of substate violence resulting in 25 battlefield deaths or more are denoted by *Y*_*t*, *i*_ [29]. Temperature and precipitation data are denoted, respectively, *T*_*t*, *i*_, *P*_*t*, *i*_ ∈ ℝ [30]. Vector *S*_*t*, *i*_ includes subnational controls. Finally, country-year fixed effects are added so that inferences are drawn from within-country comparisons as well as to minimize possible omitted variable bias not eliminated by the controls themselves. Conditional logistic regression is used so that coefficients of interest are not biased by the inclusion of fixed effects. Summary statistics for all variables are presented in Table 2.

**Table 2:** Descriptive Statistics.

Statistic	N	Mean	St. Dev.	Min	Max
Incidence of Substate Violence	52,060	0.21	0.41	0	1
Average Annual Temperature	31,360	63.85	15.29	-12.25	87.80
Total Yearly Precipitation	38,432	5,950.15	27,168.55	0.00	1,185,534.00
Province Size (sq km)	52,060	56,286.20	292,672.20	1.06	12,302,740.00
Average Ruggedness	52,060	130.58	144.25	0.00	938.69
Average Absolute Elevation	51,800	570.24	651.22	-7.82	4,881.88
Evergreen Forest (sq 300 meter)	52,060	143.90	1,075.09	0.00	40,694.14
Deciduous Forest (sq 300 meter)	52,060	47.34	166.10	0.00	3,864.83
Wetlands (sq 300 meter)	52,060	10.99	88.84	0.00	2,217.84
Croplands (sq 300 meter)	52,060	91.15	277.23	0.00	4,466.43
Number of Ethnic Groups	44,251	8.91	21.47	0	35
Distance to Foreign Country	46,960	204.67	284.24	0.00	2,960.00
Distance to Capital City	46,960	492.73	683.62	2.00	7,755.92
Refugee Camp(s) (binary indicator)	52,060	0.02	0.14	0	1
IDP Camp(s) (binary indicator)	52,060	0.00	0.02	0	1
Year	52,060	–	–	1989	2008

This analysis is then replicated for areas without economically meaningful levels of agriculture by eliminating all province-year units containing more than a single unit of croplands. Additionally, areas that contain either camps of refugees or internally displaced people are also eliminated to control for climate-induced migration [31].

A modified form of Eq (1) is tested:

$$P\left(Y_{t,i}=1|T_{t,j},P_{t,j},S_{t,j}\right)=logit^{-1}\left(\eta_{i}+\zeta T_{t,j}+\varphi P_{t,j}+\vartheta S_{t,j}+\nu_{t,k}\right)$$ (2)

where *j* ⊂ *i* and represents provinces containing no more than a single (300 × 300 meter) unit of croplands within a given year *t*.

Specifically, vector *S*_*t*, *i*_ includes land cover types conducive to insurgency violence (deciduous forest, evergreen forest, and wetlands) [28]; logarithm of average ruggedness (relief) and absolute elevation; number of excluded and total ethnic groups [32]; average distance to nearest contiguous foreign border [30]; distance to the capital city [30]; population size (log) [30]; and province size.

Because conditional logistic regression results do not include an intercept term *α* with which to generate predicted probabilities, Bayesian logistic regression is used to do so. Prior distributions for *ϕ* (where $X_{t,i}^{T}\phi =\beta T_{t,i}+\delta P_{t,i}+\xi S_{t,i}+\varrho_{tk}$) are generated with the bayesglm() package in R. For more on the prior distributions for the coefficients and intercept, see [33]. The inclusion of fixed effects within generalized linear models can, but will not necessarily, bias coefficients [34]. Logistic results generated with both country and year fixed effects are likely to suffer incidental parameters. However, Bayesian logistic regression results that include only country fixed effects (and, therefore, fewer parameters) are generally consistent with the primary results (Tables 3 and 4). Therefore, Bayesian logistics regression results are generated with country fixed effects. To test whether these results are significantly different than those generated with conditional logistic regression, each pair of regression results is compared with a Hausman test. Using a more restrictive confidence interval of 90%, in none of the six cases can the null hypothesis that the difference in model results are equal to 0 be rejected.

**Table 3:** Incidence of Substate Violence on Temperature, (Bayesian Logistic Regression).

	*Dependent Variable: Civil War Incidence*		
	(Full Sample)	(Non-Agricultural Province Subset)	(Agricultural Province Subset)
Temperature	0.011 (0.004)**	0.017 (0.010)	0.012 (0.004)**
Precipitation	0.012 (0.002)***	0.014 (0.006)*	0.013 (0.002)***
Distance to Foreign Border	−1.265 (0.270)***	−1.475 (0.885)	−1.451 (0.304)***
Distance to Capital	−0.139 (0.092)	−0.535 (0.183)**	0.063 (0.112)
Province Size	−0.002 (0.0005)***	0.001 (0.001)	−0.003 (0.001)***
Population (log)	0.142 (0.030)***	0.092 (0.083)	0.149 (0.034)***
Observations	26,477	6,802	19,489
R^2^	0.008	0.006	0.010
Max. Possible R^2^	0.327	0.274	0.336
Log Likelihood	−5,135.184	−1,066.033	−3,894.329
Wald Test (df = 13)	206.590***	37.880***	186.100***
LR Test (df = 13)	219.219***	41.711***	201.239***
Score (Logrank) Test (df = 13)	215.437***	39.379***	195.984***

*p < 0.05;

**p < 0.01;

***p < 0.001

NOTE—All regressions include terrain and ethnic group controls and country fixed effects.

**Table 4:** Incidence of Substate Violence on Temperature, Sub-Saharan/Sahelian Units Excluded (Bayesian Logistic Regression).

	*Dependent Variable: Civil War Incidence*		
	(Full Sample)	(Non-Agricultural Province Subset)	(Agricultural Province Subset)
Temperature	0.016 (0.004)***	0.031 (0.011)**	0.014 (0.005)**
Precipitation	0.013 (0.002)***	0.011 (0.009)	0.013 (0.002)***
Distance to Foreign Border	−0.976 (0.305)**	−1.777 (1.512)	−1.255 (0.331)***
Distance to Capital	−0.105 (0.104)	−0.220 (0.241)	0.072 (0.121)
Province Size	−0.004 (0.001)***	−0.009 (0.005)	−0.004 (0.001)***
Population (log)	0.162 (0.034)***	0.168 (0.115)	0.139 (0.036)***
Observations	18,021	4,643	13,301
R^2^	0.013	0.011	0.014
Max. Possible R^2^	0.343	0.258	0.362
Log Likelihood	−3,666.597	−667.721	−2,889.145
Wald Test (df = 13)	213.820***	44.290***	176.140***
LR Test (df = 13)	234.674***	52.220***	193.242***
Score (Logrank) Test (df = 13)	228.197***	47.735***	187.358***

*p < 0.05;

**p < 0.01;

***p < 0.001

NOTE—All regressions include terrain and ethnic group controls and country fixed effects.

Predicted probabilities of conflict incidence for the range of annual average temperature values observed within the 95th percentile of the study data are calculated $\mu =1/n\sum_{i=1}^{n}(e^{\left(X_{t,i}^{T}\phi \right)}/(e^{\left(X_{t,i}^{T}\phi \right)}+1))$ ∀ temperature value *t* ∈ [0°C and 29°C]. Confidence intervals at the 95% significance level are generated using quasi-Bayesian Monte Carlo simulation.

This testing strategy offers several improvements on earlier temperature-conflict research. First, a global analysis of temperature and conflict incidence for the past two decades avoids external validity concerns. Second, a battle-death threshold of 25 significantly relaxes Burke et al.’s threshold on whether violence is coded as substate conflict. Previously omitted conflict years of concern to Buhaug, such as those of Sierra Leone’s civil war, are included in the dataset.

Controlling for previously omitted terrain variables further reduces the potential for bias. Finally, adopting the province-year as the unit of analysis instead of a smaller spatial unit, while excluding areas with communities of displaced persons, reduces the likelihood that a finding of temperature-conflict correlation in non-agricultural settings is influenced by possible spillover effects.

Temperature is spatially correlated. Therefore, the model must distinguish between two possible processes that could each produce positive significant temperature-conflict associations in non-agricultural areas. First, violence could begin with a drop in agricultural output and spread to nearby non-agricultural areas. Alternatively, the source of the temperature-violence correlation observed in the non-agricultural province could be in the province itself. The former error in measurement is unlikely for two reasons. First, with the province-year as the unit of analysis, a temperature-conflict correlation in non-agricultural settings would result from spillover violence only if conflict grounded in agriculture travels over significant distances. Second, because the non-agricultural model excludes provinces with populations of displaced persons, any such spillover violence would not be the result of climate induced migration patterns. Alternative plausible mechanisms through which agriculturally induced violence might travel over long distances are not known. Aggregating to the province level does carry some risk of biasing coefficients but less so than the hitherto standard practice of aggregating to the country level.

Restricting the data verifies the model’s robustness. Provincial units from sub-Saharan and Sahelian African countries are expunged from the data. This approach ensures that results are not driven by one geographic context for which prior research suggests the effect is strongest and conforms to Buhaug’s selection of relevant African states (Table 5).

**Table 5:** Incidence of Substate Violence on Temperature, Sub-Saharan and Sahelian Provinces Excluded (Conditional Logistic Regression).

	*Dependent Variable: Civil War Incidence*		
	(Full Sample)	(Non-Agricultural Province Subset)	(Agricultural Province Subset)
Temperature	0.106 (0.013)***	0.164 (0.050)***	0.092 (0.013)***
Precipitation	0.017 (0.004)***	0.034 (0.023)	0.016 (0.004)***
Distance to Foreign Border	−0.727 (0.440)	−1.711 (3.453)	−1.504 (0.480)**
Distance to Capital	−0.001 (0.155)	−0.374 (0.507)	0.193 (0.174)
Province Size	−0.008 (0.001)***	−0.016 (0.009)	−0.007 (0.001)***
Population (log)	0.629 (0.062)***	0.895 (0.311)**	0.539 (0.067)***
Observations	18,021	4,643	13,301
R^2^	0.028	0.027	0.030
Max. Possible R^2^	0.129	0.056	0.136
Log Likelihood	−986.521	−70.923	−771.582
Wald Test (df = 13)	351.280***	52.410***	276.650***
LR Test (df = 13)	510.175***	125.639***	398.876***
Score (Logrank) Test (df = 13)	441.973***	97.974***	343.782***

*p < 0.05;

**p < 0.01;

***p < 0.001

NOTE—All regressions include terrain and ethnic group controls and country-year fixed effects.

A final threat to inference might result if particular regions differ from others in unobserved ways that are correlated with both temperature and conflict within each country. Absent a practicable method for conditional logistic regression with two-way fixed effects, a generalized linear mixed-effects model that conditions the relationship between temperature and conflict on both the province and year tests this possibility (Tables 6 and 7).

$$Pr\left(Y_{t,i}=1\right)=logit^{-1}\left(\upsilon +\gamma T_{t,i}+\zeta P_{t,i}+\psi R_{t,i}+\varsigma_{i}+\omega_{t}\right)$$ (3)

where

$$\varsigma_{i}∼N\left(0,\sigma_{i}^{2}\right)$$

$$\omega_{t}∼N\left(0,\sigma_{t}^{2}\right).$$

**Table 6:** Incidence of Substate Violence on Temperature, (Generalized Linear Mixed-Effects Regression).

	*Dependent Variable: Civil War Incidence*		
	(Full Sample)	(Non-Agricultural Province Subset)	(Agricultural Province Subset)
Temperature	0.016 (0.005)***	0.028 (0.009)**	0.014 (0.005)**
Precipitation	0.003 (0.003)	0.005 (0.006)	0.002 (0.003)
Population (log)	0.242 (0.086)**	0.091 (0.136)	0.297 (0.106)**
Constant	−11.311 (1.168)***	−8.658 (1.931)***	−12.291 (1.433)***
Observations	26,477	6,802	19,489
Log Likelihood	−6,471.313	−1,502.362	−4,882.095
Akaike Inf. Crit.	12,958.620	3,020.723	9,780.190
Bayesian Inf. Crit.	13,024.100	3,075.323	9,843.211

*p < 0.05;

**p < 0.01;

***p < 0.001

NOTE—All regressions include ethnic group controls and province-year fixed effects.

**Table 7:** Incidence of Substate Violence on Temperature, Sub-Saharan/Sahelian Units Excluded (Generalized Linear Mixed-Effects Regression).

	*Dependent Variable: Civil War Incidence*		
	(Full Sample)	(Non-Agricultural Province Subset)	(Agricultural Province Subset)
Temperature	0.026 (0.005)***	0.039 (0.012)**	0.024 (0.006)***
Precipitation	−0.003 (0.003)	−0.014 (0.009)	−0.002 (0.003)
Population (log)	0.286 (0.111)*	0.202 (0.179)	0.330 (0.138)*
Constant	−13.562 (1.536)***	−12.334 (2.560)***	−14.328 (1.898)***
Observations	18,021	4,643	13,301
Log Likelihood	−4,413.049	−939.292	−3,401.541
Akaike Inf. Crit.	8,842.097	1,894.584	6,819.082
Bayesian Inf. Crit.	8,904.492	1,946.128	6,879.047

*p < 0.05;

**p < 0.01;

***p < 0.001

NOTE—All regressions include ethnic group controls and province-year fixed effects.

The vector *R*_*t*, *i*_ includes the remaining controls that both vary across province and year: number of excluded and total ethnic groups and population (log).
